# Response of *Sthenoteuthis oualaniensis* to marine environmental changes in the north-central South China Sea based on satellite and *in situ* observations

**DOI:** 10.1371/journal.pone.0211474

**Published:** 2019-01-29

**Authors:** Jing Yu, Qiwei Hu, Danling Tang, Hui Zhao, Pimao Chen

**Affiliations:** 1 Guangdong Provincial Key Laboratory of Fishery Ecology and Environment, Scientific Observing and Experimental Station of South China Sea Fishery Resources and Environment Ministry of Agriculture and Rural Affairs, South China Sea Fisheries Research Institute, Chinese Academy of Fishery Sciences, Guangzhou, China; 2 Key Laboratory of Ocean Remote Sensing, State Key Laboratory of Tropical Oceanography, South China Sea Institute of Oceanography, Chinese Academy of Sciences, Guangzhou, China; 3 Faculty of Chemistry and Environmental Science, Guangdong Ocean University, Zhangjiang, China; University of Minnesota, UNITED STATES

## Abstract

In the South China Sea (SCS), *Sthenoteuthis oualaniensis* (*S*. *oualaniensis*) generally has the highest stock density in spring and occupies an important position in fisheries. The responses of *S*. *oualaniensis* to marine environments in the north-central SCS in spring (March to May) from 2006 to 2010 were analyzed using satellite and *in situ* observations, with generalized additive models (GAMs). A high proportion variation in catch per unit effort (CPUE) was explained by environmental variables, including sea surface temperature (SST; explaining 13.8%) and the interaction between SST and chlorophyll a (Chl-a) concentration (explaining 16.9%). SSTs within the range of 24–28°C and Chl-a concentrations within 0.10–0.35 mg/m^3^ had positive effects on *S*. *oualaniensis* CPUE, and SST within 28–29.5°Cand Chl-a concentrations within 0.05–0.20 mg/m^3^ had negative effects. In addition, the response time of the maximum standardized catch per unit effort (SCPUE) in May to the maximum Chl-a in March was approximately six ten-day time step. The higher Chl-a and smaller stock size of *S*. *oualaniensis* in early March 2008 were partly associated with climatic anomalies caused by La Niña in spring and the limitation of *S*. *oualaniensis*by low temperature in 2008. The findings in this study can help better protect and manage *S*. *oualaniensis* resources in the SCS.

## Introduction

*Sthenoteuthis oualaniensis* (*S*. *oualaniensis*) is in the family Ommastrephidae [1], is widely distributed in the tropical and subtropical areas of the Pacific Ocean and Indian Ocean, exhibits strong phototropism and is one of the major target species of large-scale light falling-net fishing in the central South China Sea (SCS) [2]. Research has shown that *S*. *oualaniensis* habitat in the SCS is concentrated in the north-central area and contains the maximum stock density in spring [3,4]. This species migrates from offshore to coastal waters in spring to breed [5,6], primarily consumes fishes, cephalopods and crustaceans in the high trophic levels of 3 and 4, and breeds from March to May [7,8]. *S*. *oualaniensis* has a short lifecycle, a rapid growth rate, and high fecundity, thus occupying an important position in the marine ecosystem of the SCS [8].

*S*. *oualaniensis* is one of the major species in the SCS, with the number caught by the automatic squid jigging gear on the west coasts of Philippines of at a water depth of 50–100 m ranging from 0.25–9.11 squids/line hour [9–11]. In Vietnamese waters, *S*. *oualaniensis* is found at night in 18–30°C water at a depth from 125 m to the surface; the central habitat of this species is located at 14°N, 112°E (9.11 squids/line hour), where offshore upwelling exists [10,11]. In the north-central SCS, studies on *S*. *oualaniensis* have focused on its biological characteristics [12,13], yield, exploitation status [8,14,15], and environmental characteristics [3,5]. The suitable sea surface temperature (SST) of the *S*. *oualaniensis* habitat was found to be 25.6–29.6°C and the optimum SST was 28.5–29.5°C in spring [5,6,8]. These studies focused on the impacts of a single environmental factor on the distribution of *S*. *oualaniensis*, but multifactorial interactions and the weights of each factor remain unclear. In addition, the extent to which marine environments influence the distribution of *S*. *oualaniensis* habitat in the north-central SCS has rarely been reported.

As squid are short-lived ecological opportunists, the distribution and abundance of their stocks are extremely sensitive to changes in environmental conditions [13]. The distribution of habitat and abundance of *S*. *oualaniensis* are closely related to SST, Chl-a, sea surface height, the occurrence of El Niño and La Niña events [16]. Large-scale environmental variability might lead to significant fluctuations in the spatiotemporal distribution of habitat and squid stock level, especially when anomalous environmental conditions occurred. For example, Yu et al. suggested that the La Niña event resulted in more favorable habitats for *Ommastrephes bartramii* in the Northwest Pacific [17]. Nigmatullin et al. found that the El Niño phenomenon resulted in a reduced population size of *Dosidicus gigas* and subsequently a large decline in fishery yields [18]. Hu et al. concluded that the changes fishing grounds of tuna are strongly correlated with the occurrence of La Niña and El Niño events in the Western and Central Pacific, and the index of La Niña and El Niño can be used to predict fishing grounds at the year and month scales [19]. To understand the impacts of the abundance of *S*. *oualaniensis* on ocean ecosystems, especially in the north-central SCS, it is important to analyze the spatiotemporal distribution of SST, Chl-a and catch per unit effort (CPUE) in the area influenced by La Niña, as well as the mechanism of these changes.

The north-central SCS, with a water depth of more than 1000 m, is far from land; therefore, survey data are difficult to obtain. Satellite remote sensing can provide overall and spatiotemporal information about sea surface wind (SSW), SST, and Chl-a concentration, which the limited number of ship stations and surveys cannot provide [20–22]. This study analyzed the spatiotemporal variability and its possible mechanism in *S*. *oualaniensis* habitat in the north-central SCS in spring with satellite remote sensing and *in situ* investigations. The results of this study will help better protect *S*. *oualaniensis* resources and provide an ecosystem-based approach to *S*. *oualaniensis* management in the SCS.

## Materials and methods

### Research area

**Fig 1:**
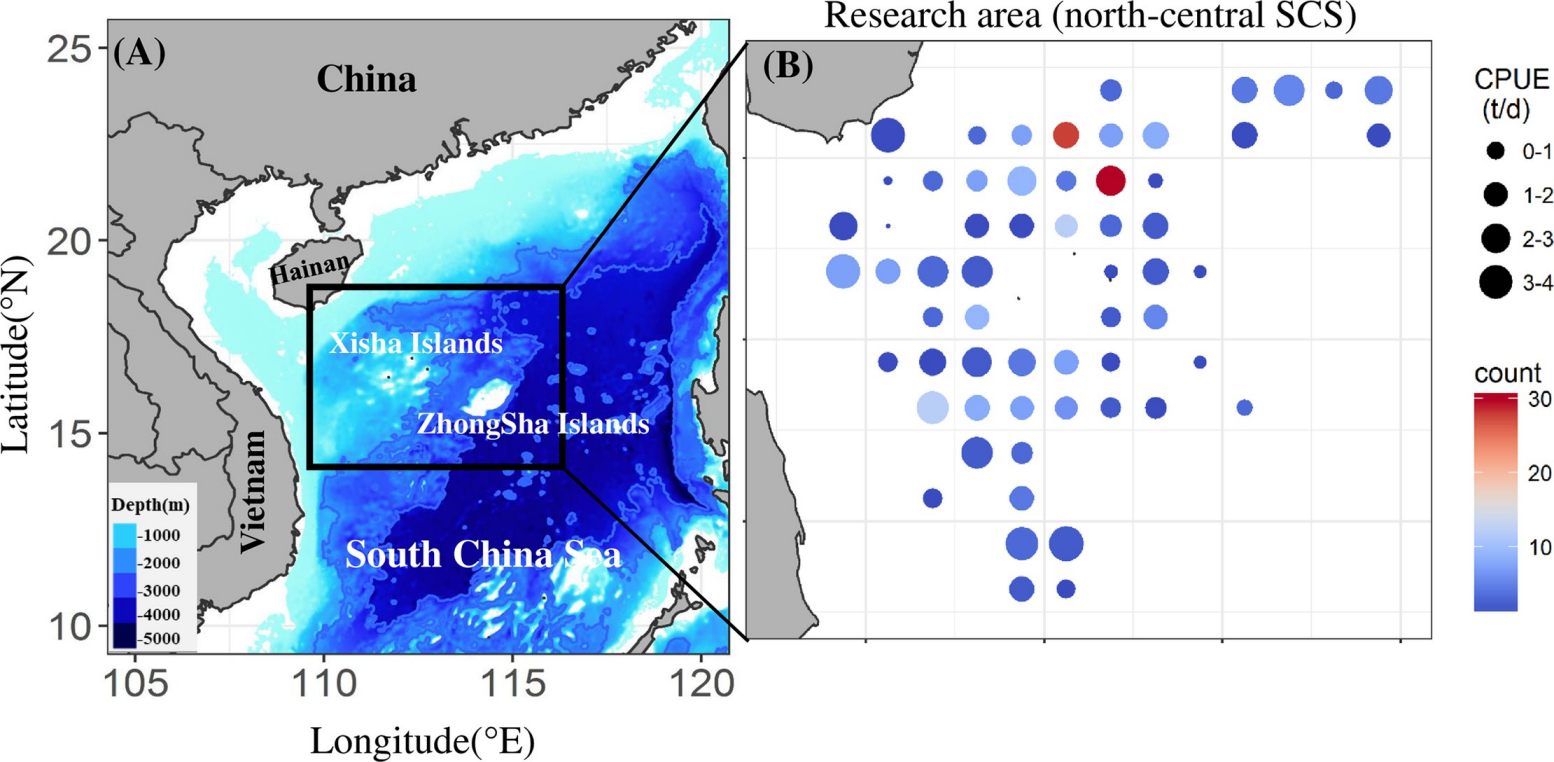
Research area. (A) Location of the north-central SCS (indicated by the black box). (B) Sampling stations and CPUE of *S*. *oualaniensis* (indicated by the size of the circle).

The north-central SCS, located at 13–19°N, 110–116°E (black box in Fig 1), including the Xisha-Zhongsha deep waters, is influenced by the South Asia monsoon [23]. The area has favorable climatic conditions with abundant marine organisms near the islands and is one of the major tropical fishing grounds [24]. *S*. *oualaniensis* data were derived from monitoring records of the large-scale light falling-net fishing ship “Qiongwenchang 33180”. Specifications of the ship are as follows: the material is wood, full length is 32 m, molded breadth is 5.8 m and molded depth is 2.9 m; there is one main engine with 220.5 kW power and one auxiliary engine with 183.8 Kw power. The fishing ship is equipped with 238 metal halide fishing lamps (×1 kW), the outboard effective length of the jackstay is 26.8 m, the ground rope length of fishing net is 208 m, the straightened net height is 66 m and the shallowest operating water depth is 40 m. The Ministry of Agriculture and Rural Affairs, Chinese Government, granted a research permit for the Xisha-Zhongsha Expedition to work within the whole sea area. The research area is not privately owned or protected, and *S*. *oualaniensis* is not protected species. No permission was required to collect the fishery data in the Xisha-zhongsha waters.

### Catch and effort data

Daily catch and effort data of *S*. *oualaniensis* were obtained from the net-cap fishing ship (Table 1). All the data were grouped by 0.5°× 0.5° grid cells (Fig 1B). The survey stations and time are shown in Table 1, including the operating date, voyage number, longitude, latitude, and yield. The CPUEof a fishing grid of *S*. *oualaniensis* was calculated as follows:

$$\mathrm{CPUE}=\frac{\sum \mathrm{Catch}}{\sum \mathrm{Fishing}\;\mathrm{days}}$$ (1)

where, the summed catches and fishing days were obtained for all the fishing vessels within a fishing grid. Ten days was chosen as the time for grouping CPUE values within each grid.

**Table 1:** Number of voyage and net of large-scale light falling-net.

Year	Month	Net time	Number of voyage
**2006**	March	99	18、19、20
	April	145	20、21、22
	May	120	22、23
**2007**	March	59	18、19
	April	79	20
	May	136	21、22
**2008**	March	67	14、15
	April	102	15、16、17
	May	75	17、18、19
**2009**	March	52	21
	April	66	22
	May	4	23
**2010**	March	86	19
	April	80	21、22
	May	48	23

### Environmental data

Satellite remote sensing SST and Chl-a data were derived from the MODIS Aqua products of NASA (http://oceancolor.gsfc.nasa.gov), with a temporal resolution of one day and a spatial resolution of 4 km. SSW data from 2006–2009 were derived from the QuikSCAT products. As The QuikSCAT sensor had not been in service since 2009, the SSW data in 2010 were derived from WindSat level-3 products (http://www.remss.com). All SSW data were ascending orbit data with a temporal resolution being day and spatial resolution of 0.25° × 0.25°.

### Generalized additive model fitting procedures

General additive models (GAMs) [25] were used to investigate the influence of environmental variables on the abundance and distribution of fishery resources. This flexible class of mathematical models allows the incorporation of smoothing functions to model the nonlinear effect of continuous explanatory variables [26]. GAMs were constructed in R (Version 3.3.0) (R development Core Team, 2016), using the GAM function of the mgcv package [25], with CPUE as the response variable and time (year and month), location (latitude and longitude) and environmental (SST and Chl-a) variables as the explanatory variables. The formulation of this model was as follows:

Log(CPUE+1)=s(Year)+s(Month)+s(Latitude)+s(Longitude)+s(SST)+s(Chl−a)+s(SST,Chl−a) (2)

where, s(x) denotes a spline smoothing function of the covariate x or the interaction between two covariates. SST and Chl-a were treated as interaction terms to account for interactive effects between these drivers of variation in the species, probably driven by marine environmental variables. Logarithmic transformation of the CPUE was used to normalize its asymmetrical frequency distribution, and a value of one was added to all CPUE values to account for zero-value CPUE data. A Gaussian model with an identity link function was the most appropriate and reliable fit for the transformed CPUE data compared with models with other possible GAM error distributions and link functions [27,28].

The modeling approach, based on information theory [29], was chosen to build sets of candidate models of increasing complexity and to select the best model based on minimizing an information criterion. In all cases, decreasing the generalized cross validation (GCV) score coincided with decreasing values of Akaike’s information criterion (AIC) and increasing percentages of explanatory deviance [29]. The best GAM was obtained with a backward stepwise procedure by selecting significant P values for each variable. The significance of the factor and the nonlinear contribution of the factor to the nonparametric effect were evaluated by an F test and a chi-square test, respectively [27–29].

### Data processing

MATLAB 2015b software was used to read the satellite remote sensing SST, Chl-a and SSW data in the research area, and invalid values were eliminated. To reduce the impact of the lack of fishery data in this study, all environmental variables had a basic time unit of 10 days. The mean value of factors was also computed over a time unit of 10 days being the unit, consistent with the CPUE time scale. GrADS software was used to draw spatiotemporal distribution diagrams of SSW, SST, Chl-a and standardized CPUE (SCPUE).

## Results

### CPUE standardization

**Fig 2:**
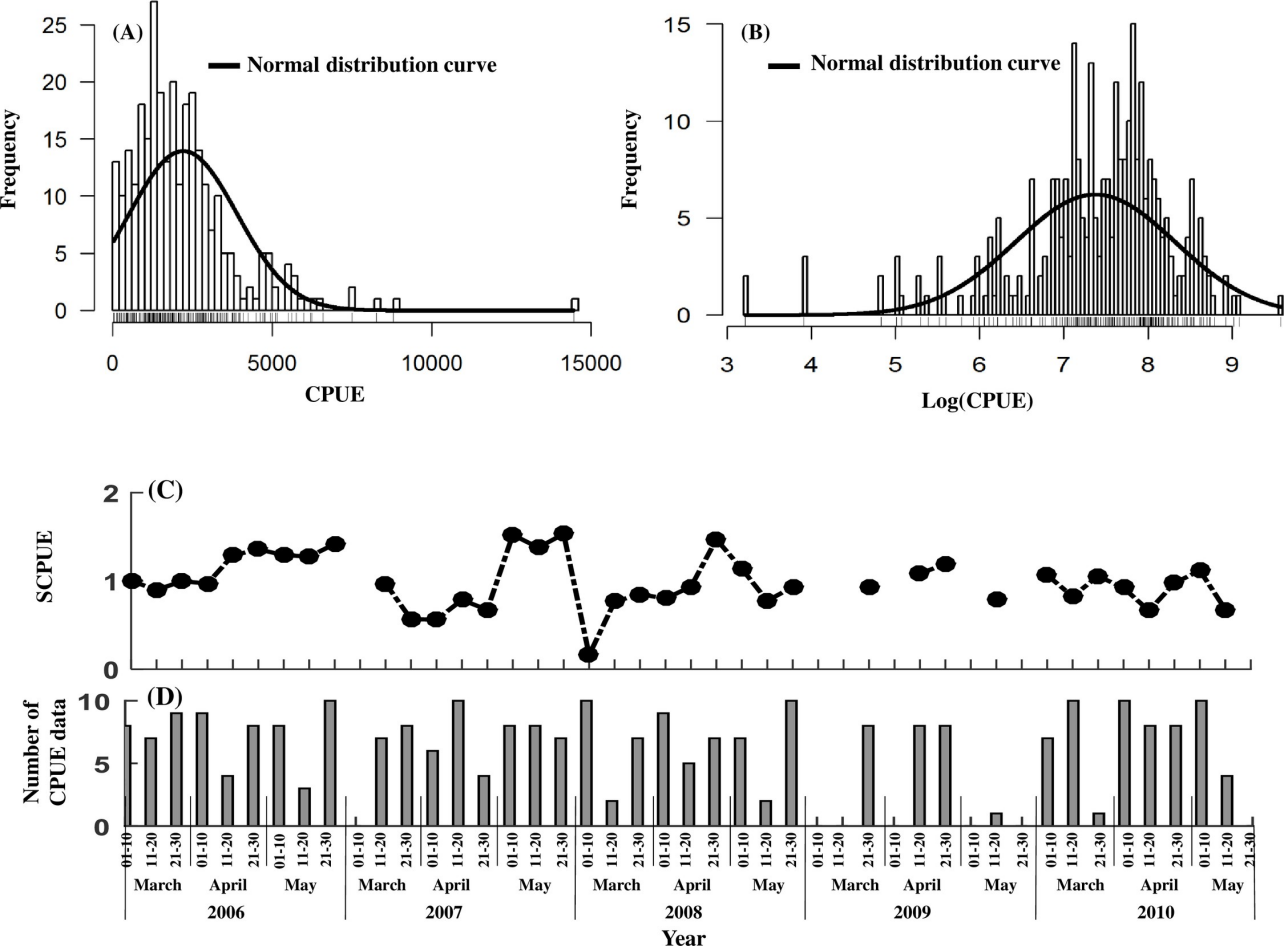
CPUE standardization. (A) Distribution of CPUE frequencies. (B) Distribution of logarithmically t transformed CPUE frequencies. (C) SCPUE. (D) Number of *S*. *oualaniensis* survey data points.

Before GAMs were used to standardize the CPUE of *S*. *oualaniensis*, it was necessary to determine the statistical distribution of this variable. The histogram of CPUE presented a partially normal distribution (Fig 2A). After logarithmic transformation of CPUE, its partially normal distribution was improved and generally was consistent with a normal distribution (Fig 2B). Therefore, logarithmic transformation of CPUE was conducted before standardization. Based on the GAM, the SCPUE increased overall from March to May, with fluctuations during 2009–2010 (Fig 2). The SCPUE varied from 0.50–1.50 during 2006–2010, with a maximum of 1.60 in late May 2007 and a minimum of 0.16 in the first ten days of March 2008 (Fig 2C).

During the investigated period, the number of data points for *S*. *oualaniensis* in each period of ten days was usually 5–10. The numbers of points in the middle of March and May 2008 and in late March 2010 were relatively small, and some data during March-May 2009 were missing (Fig 2D).

### GAM analysis

**Fig 3:**
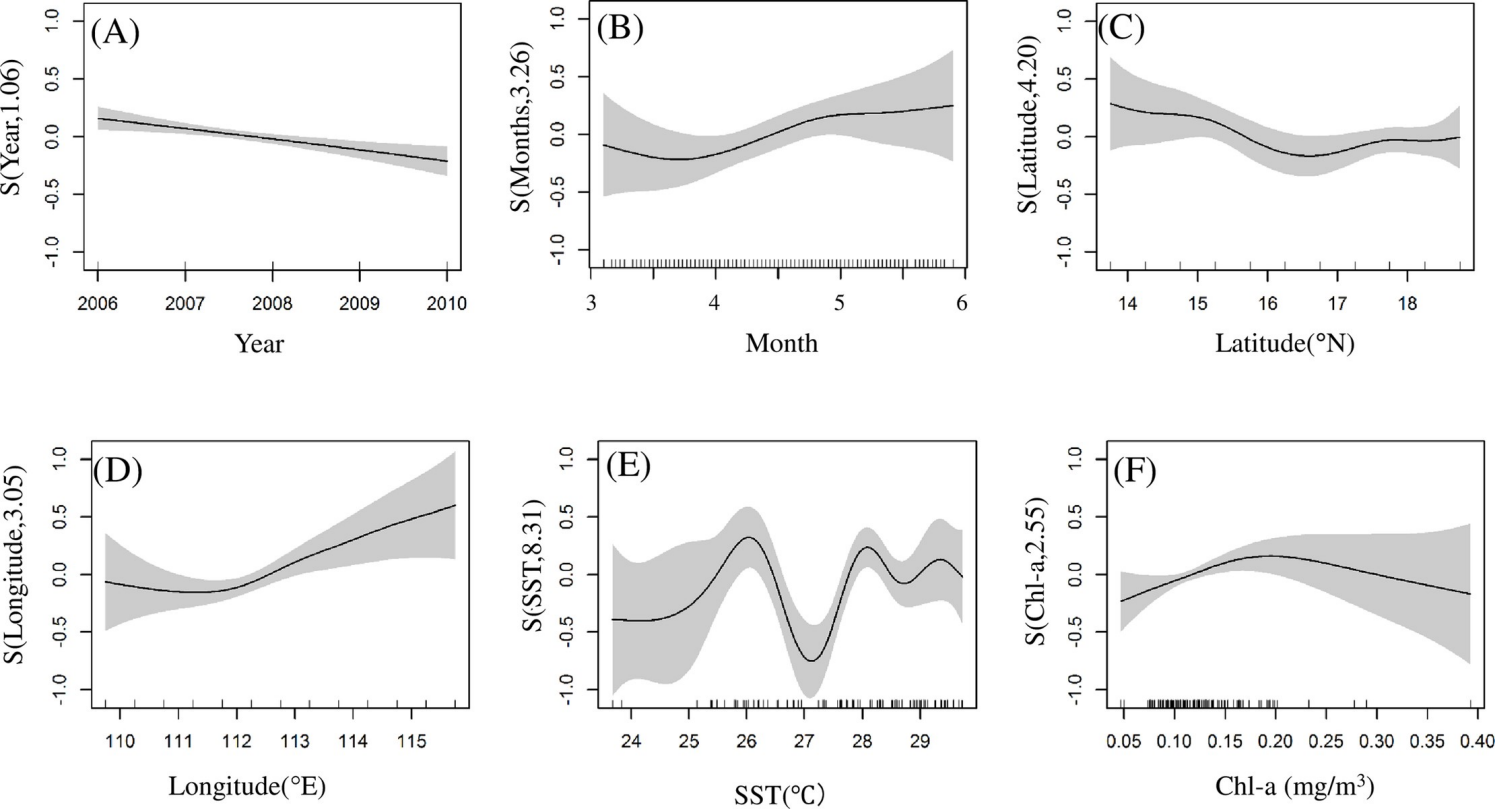
Effects of spatiotemporal and environmental factors on *S*. *oualaniensis* CPUE based on GAMs. (A) Year, (B) month, (C) latitude, (D) longitude, (E) SST, and (F) Chl-a. Shaded regions indicate the 95% confidence intervals. Rug plots on the x-axis indicate the relative density of data points.

The results of GAM fitting indicated that the best model for *S*. *oualaniensis* included six explanatory variables (Table 2, Fig 3). The deviance explained by this model was 59.9%, with an R^2^ of 0.48 (Table 2). The relationship between CPUE and each variable in the GAM allowed us to examine the contribution of each variable separately (Table 3). For *S*. *oualaniensis*, variables accounting for the greatest deviance in the univariate GAM analysis were the interaction between SST and Chl-a (explaining16.9%), SST (explaining13.8%) and longitude (explaining 8.7%). As indicated by the ANOVA F-ratio test, all the factors in the model were significant (P < 0.05), except for year. When year was added to the model, the AIC and GCV values of the model continued to decrease, and the cumulative deviation interpretation of the model increased, indicating an increase in the fit and generalization of the model. Therefore, year was kept in the model. The chi-squared test indicated nonparametric smoothing effects of the predictive variables. The most significant effect in the nonparametric smoothing was that of the interaction between SST and Chl-a, as indicated by the chi-square test values of each predictive variable shown in Table 3.

**Table 2:** Analysis of deviance for generalized additive model (GAM) fitted to the CPUE.

Model factors	Residual deviance	R^2^ value	AIC value	GCV value	Deviance explained(%)
**Log(CPUE+1)** **= NULL**	124.14	0.00	455.21	0.68	0.00
**Log(CPUE+1)** **= s(Year)**	120.46	0.02	454.19	0.67	2.97
**Log(CPUE+1)** **= s(Year) + s(Month)**	112.44	0.07	447.21	0.65	9.43
**Log(CPUE+1)** **= s(Year) + s(Month)+s(Lat)**	107.29	0.10	442.31	0.64	13.60
**Log(CPUE+1)** **= s(Year) + s(Month)+s(Lat)+s(Lon)**	96.43	0.16	436.45	0.61	22.30
**Log(CPUE+1)** **= s(Year) + s(Month)+s(Lat)+s(Lon)+s(SST)**	79.31	0.30	406.79	0.53	36.10
**Log(CPUE+1)** **= s(Year)+s(Month)+s(Lat)+s(Lon)+s(SST)+s(Chl-a)**	70.73	0.34	401.69	0.51	43.00
**Log(CPUE+1)** **= s(Year)+s(Month)+s(Lat)+s(Lon)+s(SST)+s(Chl-a)+s(SST,Chl-a)**	49.91	0.48	370.23	0.46	59.90

**Table 3:** The contribution of selected environmental variables in the GAM.

Variables	d.f.	Contribution(%)	Pr(*F*)	Pr(*Chisq*)
Year	2.28	1.97	0.080	0.070
Month	2.88	6.46	0.005	0.004
Latitude	1.88	4.17	0.013	0.010
Longitude	6.94	8.70	0.013	0.007
SST	3.25	13.80	0.000	0.000
Chl-a	1.92	6.90	0.010	0.020
SST:Chl-a	17.09	16.90	0.000	0.000

Relationships between CPUE and the explanatory variables from GAMs showed that the highest *S*. *oualaniensis* CPUE was in May (Fig 3). A significant increase was observed in the period from March to May, and the maximum was reached in May (Fig 3B). Interannual patterns showed a linear decrease in abundance from 2006 to 2010 (Fig 3A). In terms of spatial factors, the CPUE slowly decreased from 14–18°N (Fig 3C). Longitude and *S*. *oualaniensis* CPUE showed a negative linear relationship at 110–112°E, and a positive linear relationship at 112–116°E (Fig 3D). SST and *S*. *oualaniensis* CPUE increased from 24°C to 29°C, declined sharply at 27°C, and peaked at 26°C (Fig 3E). The effect of Chl-a on *S*. *oualaniensis* CPUE increasedfrom 0.05–0.20 mg/m^3^and decreased from 0.20–0.40 mg/m^3^ (Fig 3F).

#### Interaction effect of SST, Chl-a and SCPUE

**Fig 4:**
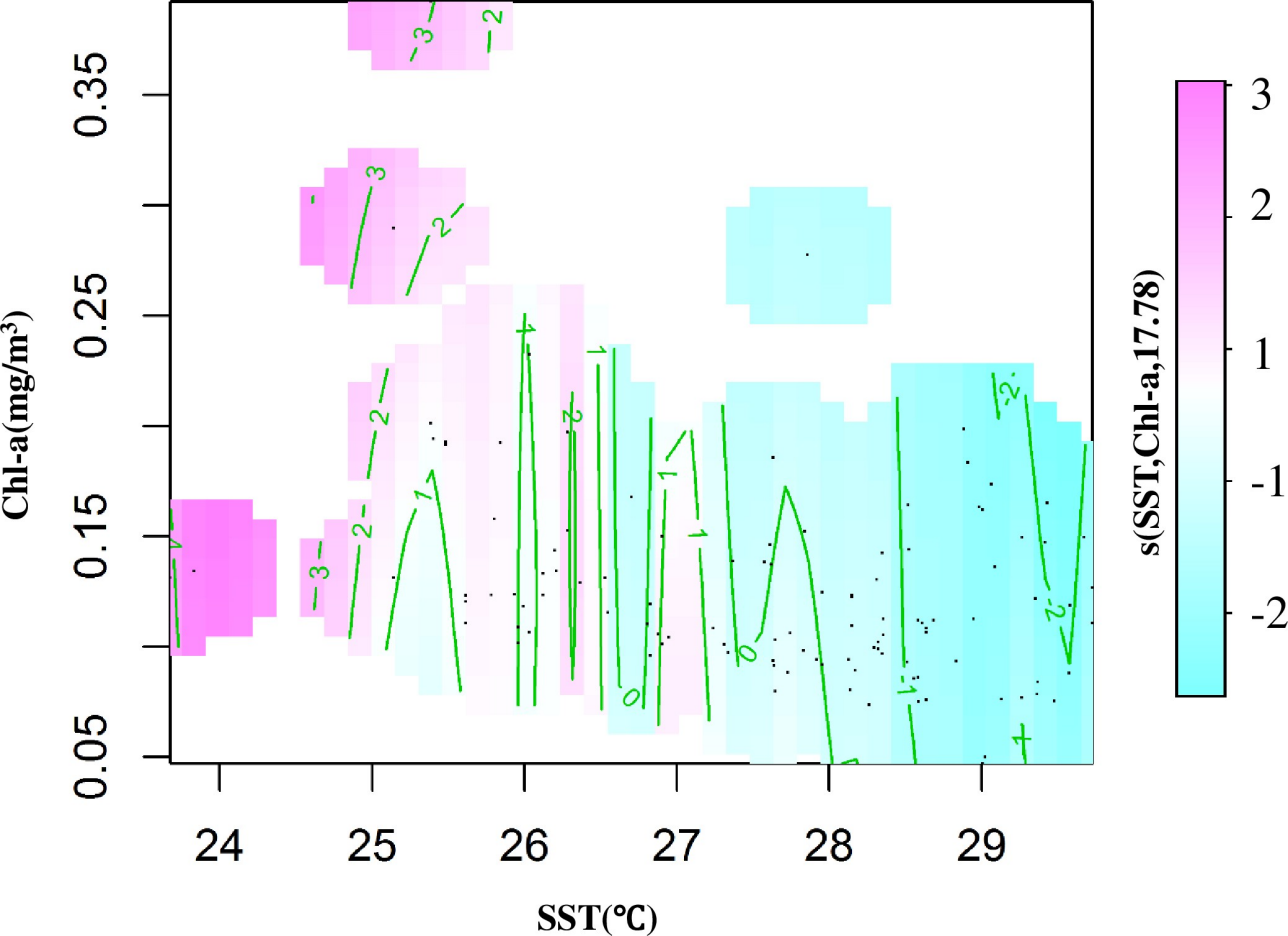
Interaction effects of *S*. *oualaniensis* CPUE, SST and Chl-a based on GAMs.

**Fig 5:**
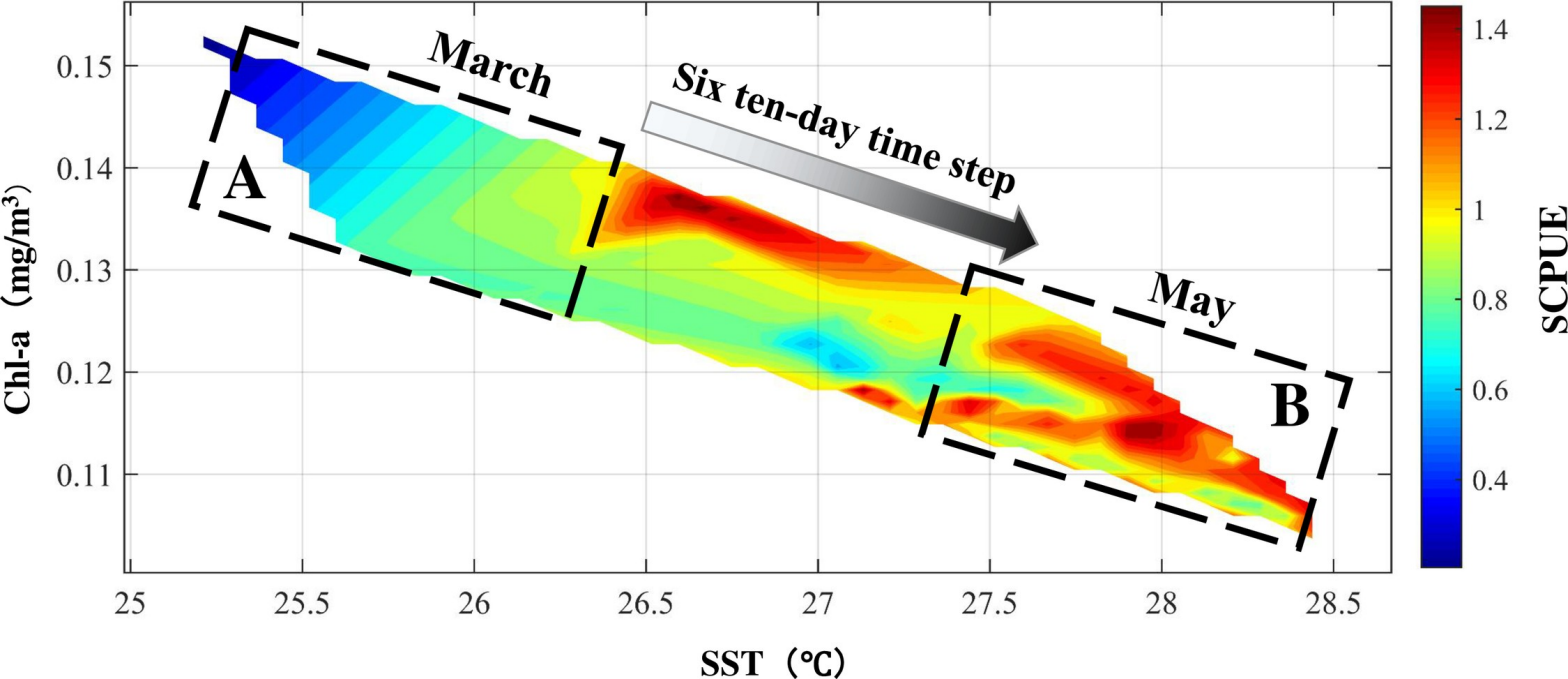
Relationship among SST, Chl-a and SCPUE. Boxes A and B indicate the phenomena of “low SST, high Chl-a and low SCPUE” in March and “high SST, low Chl-a and high SCPUE” in May, respectively.

The interaction effect of SST and Chl-a on the SCPUE was analyzed by GAMs, which revealed a positive effect when SST was24-27°C and Chl-a was0.10–0.35mg/m^3^, and a negative effect when SST was28-29.5°C and Chl-a was 0.05–0.20 mg/m^3^ (Fig 4). The interaction effect on SCPUE decreased gradually with increasing SST and decreasing Chl-a (Fig 4). Analysis of spatial trend surface interpolation showed effects of SST and Chl-a on *S*. *oualaniensis* SCPUE (Fig 5). In the ranges of 25–28.5°C and 0.10–0.16 mg/m^3^ Chl-a, the SCPUE increased slowly with an increase in SST and a decrease in Chl-a, reaching its maximum in May. In addition, the maximum SCPUE and Chl-a appeared in May and March, respectively, suggesting that the response of *S*. *oualaniensis* SCPUE to Chl-a may have lagged by approximately six ten-day time steps (Fig 5).

### Spatiotemporal variations in SSW, SST, Chl-a and SCPUE

**Fig 6:**
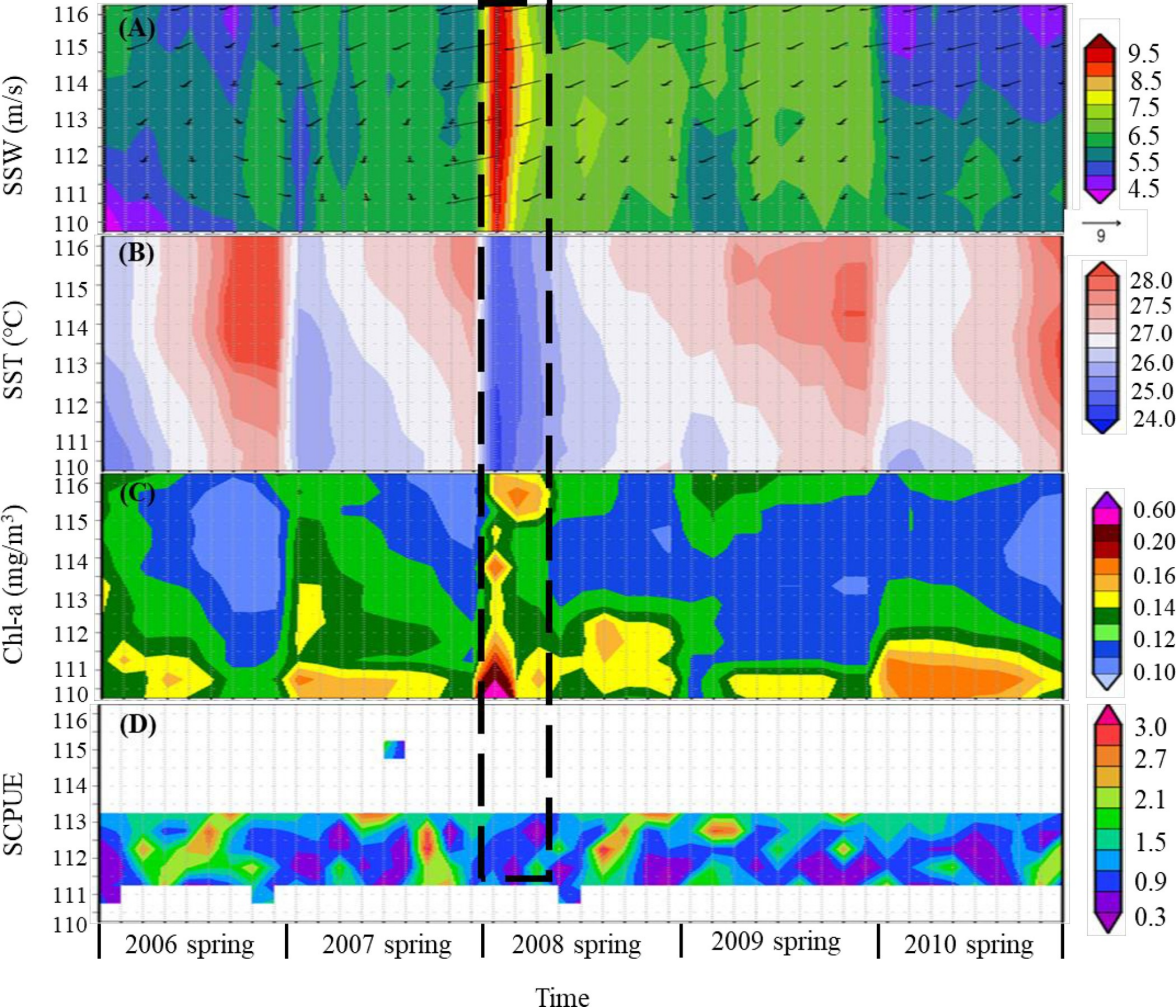
Time series analysis of SSW (A), SST (B), Chl-a (C), and SCPUE (D). The dashed box shows the influence of climate anomalies on *S*. *oualaniensis* SCPUE in 2008.

The longitudinal distribution of SSW varied within 5–6 m/s (Fig 6A). The maximum SSW was 9.2 m/s in early March 2008. From March to May 2008, the SSW decreased and fluctuated within 6–9 m/s. In addition, the SSW in 2008 was higher than that in 2006–2007 and that during 2009–2010 (Fig 6A).

The longitudinal distribution of SST progressively increased. In late May 2006, the SST was the highest, at 28.5°C. In early March in 2008, the SST was the lowest, at 25.1°C During 2006–2008, the SST increased rapidly within 26–28.5°C, while during 2009–2010, it changed relatively slowly within 27–28.5°C. In early March 2008, the SST was 25.1°C, which was lower than that observed within the same periods of other years (Fig 6B).

Chl-a decreased gradually within the longitudinal range of 110–116°E but remained at a high level. Chl-a was highest within 110–111.5°E in early March 2008, at 0.15 mg/m^3^, and was lowest within 113–114.5°E in late May 2010, at 0.10 mg/m^3^. Within 110–116°E, Chl-a decreased rapidly from March to May during 2006–2008 within 0.10–0.16 mg/m^3^ and changed relatively little during 2009–2010 within 0.10–0.14 mg/m^3^ (Fig 6C).

Within the longitudinal range of 111.5–113°E, the SCPUE gradually increased. The SCPUE was the highest in late May 2008, at 2.85, and was lowest in early March 2008, at 0.16 (Fig 6D). The SCPUE increased rapidly within 0.10–3.00 during 2006–2008 and increased slowlyduring 2009–2010 (Fig 6D).

**Fig 7:**
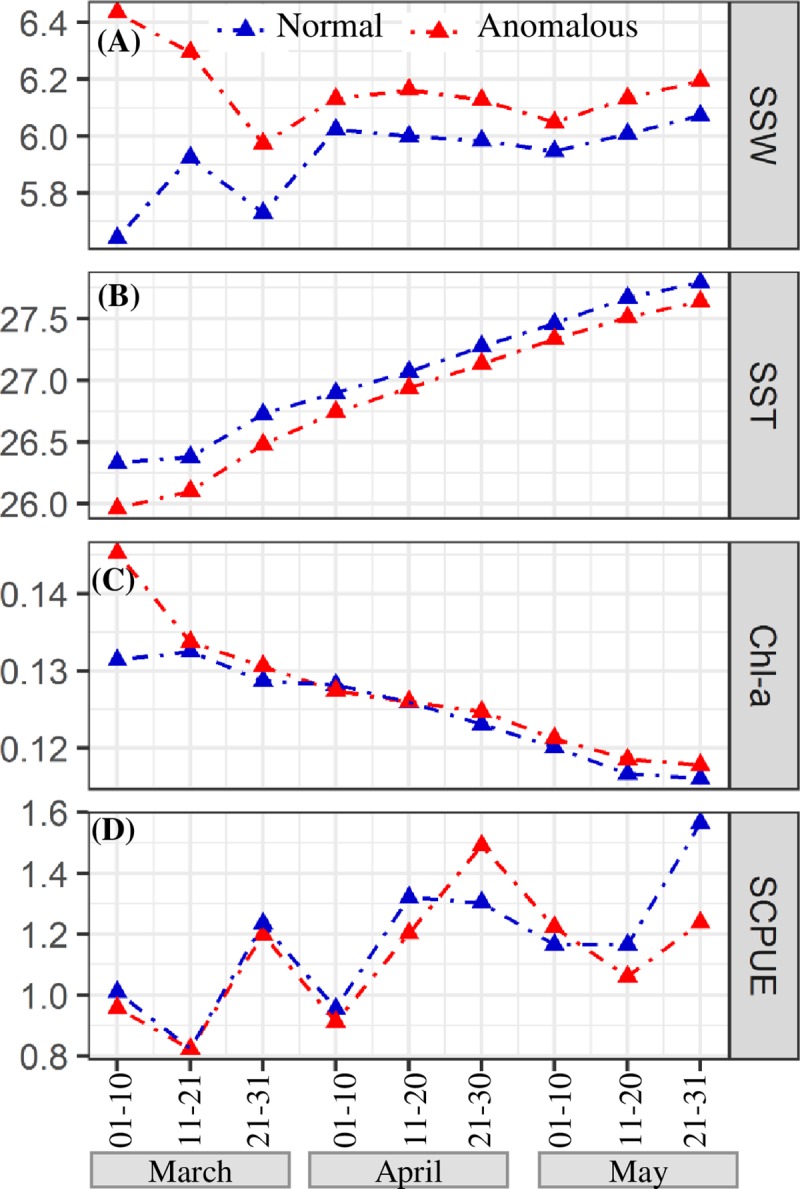
Climatological average of SSW (A, m/s), SST (B, °C), Chl-a (C, mg/m^3^), and SCPUE (D) from March to May during the anomalous (2007–2010, red dotted line) and normal (2006–2007 and 2009–2010, blue dotted line) periods.

The climatological averages of SSW, SST, Chl-a and SCPUE in normal years (2006–2007 and 2009–2010) and anomalous year (2008) are shown in Fig 7. In the anomalous year (2008), changes in the marine environment (SSW, SST, Chl-a) were greater than those in normal years (2006–2007 and 2009–2010), especially in early March (Fig 7). Specifically, SSW and Chl-a were significantly higher (Fig 7A and 7C) and SST was lower than those in normal years from March to May (Fig 7B). Furthermore, in the anomalous year (2008), the SCPUE was slightly lower in March and increased significantly in late April (Fig 7D).

## Discussion

The landing statistics of *S*. *oualaniensis* was used as a proxy for their abundance in the north-central SCS. The index of CPUE is generally assumed to be proportional to stock size [17]. However, CPUE data can be variable or compromised by environmental changes or management through time. In addition, changes in the abundance of pelagic species from commercial data were often difficult because of the high variability of these resources and their extremely aggregated geographical distribution [16]. However, as *S*. *oualaniensis* is the major target species of the north-central SCS and catches are not restricted by management strategy, the CPUE was considered as a proxy of their abundance in the resrarch area. By matching catch statistics with environmental factors, this study provided new insight into the response of *S*. *oualaniensis* to marine environmental changes in the north-central SCS.

### SST effects on *S. oualaniensis*

Temperature is one of the major environmental factors affecting squid activities, including aggregation, breeding, and emigration [19, 30]. *S*. *oualaniensis* is a warm-water oceanic squid species with a habitat closely associated with SST, and the ability to adapt to SST [12, 16]. This research indicated that the SST of *S*. *oualaniensis* habitat was 25–28.5°C in the north-central SCS in spring (March-May), and a large number of *S*. *oualaniensis* individuals appeared in the area with an SST of 26.5–28.5°C (Figs 2E, 5 and 6). In the south-central SCS, the SST of *S*. *oualaniensis* habitat was 25.6–29.6°C in spring, and the most appropriate SST was 28.5–29.5°C [6]. Exploratory fishing production showed that the appropriate SST of the *S*. *oualaniensis* central habitat was within 27–29°C in the northern Arabian Sea [31] and was within 25–29°C in the northwest Indian Ocean [32,33]. The appropriate SST of *S*. *oualaniensis* habitat varied among watersbecause the latitude of cental *S*. *oualaniensis* habitat was different and the appropriate SST of *S*. *oualaniensis* was higher compared to those of other developed cephalopods [6]. In this study, the research area was at a latitude similar to that of the investigated area in the northern Arabian Sea; therefore, the appropriate SST for *S*. *oualaniensis* was consistent with that in the Arabian Sea.

CPUE standardization based on GAMs reflect variations in *S*. *oualaniensis* resources more objectively [34] and also allows the influences of different spatiotemporal and environmental factors on CPUE to be measured [35]. In this research, SST explained more deviance in the CPUE, reaching 13.80% of deviance explained (Table 3). Thus, *S*. *oualaniensis* was greatly influenced by water temperature [6, 8, 30]. As low SST in March was inappropriate for the growth of *S*. *oualaniensis*, the species entered the peak breeding season in April [3], when water temperature rose gradually, and higher net primary production (NPP) occurred during March-April. Larvae and juvenile fish of *S*. *oualaniensis* experienced a suitable growth environment, and the number of individuals in April and May gradually increased due to theirshort life cycle, fast growth and breeding migration, peaking in May (Figs 3,6, and 7). The total accumulative deviation explained by theGAM was 59.90% (Table 2), which was similar to previous results [36–38]. The GAM had limited ability to explain deviance for *S*. *oualaniensis* CPUE, mainly because the growth of *S*. *oualaniensis* is influenced by multiple factors, including water temperature, currents, nutrients, and prey biomass [3, 31, 39]. Environmental factors obtained by satellite remote sensing have limited ability to explain the abundance of *S*. *oualaniensis* (Tables 2 and 3). Moreover, the number of survey data points affects model calculation and statistical analysis (Table 2, Fig 2). In follow-up studies, factors such as ship type, salinity, currents, water depth, etc. will be considered to improve model accuracy.

### Chl-a effects on *S. oualaniensis*

Chl-a can reflect the standing crop of phytoplankton, which is the most important constituent of NPP [1, 40]. This measure also directly reflects the biomass and distribution of zooplankton, which are closely related to the distribution of the central habitat of *S*. *oualaniensis* [39, 40]. GAM analysis showed that Chl-a had secondary significance to *S*. *oualaniensis* CPUE (Table 3). This result occurred because *S*. *oualaniensis* primarily consumes fishes, cephalopods and crustaceans, with high trophic levels of 3 and 4 [3,7,8], rather than directly feeding on phytoplankton. The effect of Chl-a on the spatiotemporal distribution of *S*. *oualaniensis* was indirect and delayed [8, 41]. In the north-central SCS, the phenomena of “high Chl-a and small stock size” and “low Chl-a and large stock size” in spring were observed (Fig 5), partly related to the delay in stock growth caused by the biological habit of *S*. *oualaniensis* [12]. This species has a short lifecycle, with the maximum age of larva and juvenile squid being 100–110 days [41], and enters the peak breeding season for the growth of larva and juvenile squid in April [7]. Chl-a was at a high level in March, providing prey allowing an increase in *S*. *oualaniensis* resources from April to May (Figs 6 and 7). However, *S*. *oualaniensis* migrated from the offshore area to the northern shallow waters of the SCS to breed in spring [6, 42]. Water temperature gradually increased, reaching the optimum habitat temperature for *S*. *oualaniensis* and the multi-island topography (Xisha-Zhongsha Islands) provided favorable conditions for the breeding migration and larvae aggregation of *S*. *oualaniensis* in the north-central SCS [43,44].

Larval and juvenile *S*. *oualaniensis* grew rapidly with high primary production and appropriate water temperature, resulting inthe appearance of maximum stock size in May (Figs 5, 6, and 7). The delay between the peak value of *S*. *oualaniensis* and that of Chl-a was approximately six ten-day time steps (Fig 5). In addition, the SCPUE of *S*. *oualaniensis* exhibited small fluctuations during 2009–2010, which might have been related to the small number of survey data pointsin this period (Fig 2C). Further investigation will be conducted to improve the size of the survey dataset.

### Low *S. oualaniensis* density induced by climatic anomalies in 2008

Climatic anomalies, such as El Niño and La Niña, influence the distribution and stock density of fishery resources [19]. As *S*. *oualaniensis* is a squid with a short life cycle, its abundance was extremely vulnerable to anomalous environmental conditions. SSW was at its maximum in early March 2008, when SST was the lowest (expressed by dotted boxes in Fig 6), which might have been related to the La Niña event in early 2008 and local circulation anomalies in the north-central SCS [45, 46]. The La Niña event formed in August 2007 and ended in April 2008, during which time sea temperature in the middle-eastern equatorial Pacific Ocean and the SCS was partially low [45]. In addition, the tropical convective activity in the Western Pacific warm pool in spring of 2008 was strong, and the north-central SCS waters are located at the edge of the Western Pacific warm pool [42,47]. Severe convective weather resulted in increased wind speed, decreased water temperature and large amounts of rainfall in the observation area [47,48]. La Niña increased upwelling and SSW in the Western Pacific warm pool (Fig 6), resulting in adifference in the heat distribution at the sea surface and an increase in vertical seawater exchange in this area [48,49]. As a result, nutrients from bottom water were brought to the surface. In tropical area, this bottom water with low temperature and large amounts of nutrients will increase surface Chl-a and local alga blooms (Fig 6) [47–49]. Therefore, the breeding, growth and migration of *S*. *oualaniensis* might have been limited by the lower SST in early March 2008 [50], resulting in the appearance of the lowest SCPUE observed in the five years (March-May during 2006–2010; Fig 2C). The high Chl-a in this period provided high primary production for the growth of juvenile *S*. *oualaniensis* (Fig 6), promoting the maximum abundance of *S*. *oualaniensis* in May, 2008 (Fig 2C).

### Conclusions

In this research, satellite remote sensing data of SSW, SST, Chl-a and fishery resource production were used to analyze the relationship between *S*. *oualaniensis* and marine environments in the north-central SCS in spring.

1. Positive effects on *S*. *oualaniensis* CPUE were observed for SSTs of 24–28°Cand Chl-a concentrations of 0.10–0.35 mg/m^3^, and negative effects were observed for SSTs of 28–29.5°C and Chl-a concentrations of 0.05–0.20 mg/m^3^.
2. The SCPUE of *S*. *oualaniensis* gradually increased from March to May, reaching its maximum in May. The response time of maximum SCPUE to maximum Chl-a was approximately six ten-day time steps in the north-central SCS in spring.
3. The higher Chl-a and smaller stock size of *S*. *oualaniensis* in early March in 2008 were caused by higher SSW and lower SST, which were partly associated with climatic anomalies induced by La Niña in spring 2008.

## References

[1] Diekmann R, Piatkowski U, Schneider M. Early life and juvenile cephalopods around seamounts of the subtropical eastern North Atlantic: Illustrations and a key for their identification. Institut. Fur. Meereskunde. 2002; 326:1–44.

[2] Yang DK. The resources and its exploitation and utilization of two species of squid. Journal of Shanghai Fisheries University. 2002; 11:176–179. (in Chinese with English Abstract)

[3] Fan JT, Feng X, Qiu YS, Huang Z, Chen GB. Review on the biology of purpleback flying squid in South China Sea. Journal of Guangdong Agricultural Sciences. 2013; 40:122–128. (in Chinese with English Abstract)

[4] Li B, Cheng GB, Guo Y, Chen ZZ, Zhang J, Wang DG. Hydroacoustic assessment of spatial-temporal distribution and biomass of fishery resources in the central South China Sea. South China Fisheries Science. 2016; 12: 28–37. (in Chinese with English Abstract)

[5] Yan YR, Feng B, Lu HS, Lai JY, Du SQ. Fishery biology of purpleback flying squid *Sthenoteuthis oualaniensis* in northern sea areas around nansha islands in summer. Oceanologia Et Limnologia Sinica. 2012; 32:1177–1185.

[6] Yan L, Zhang P, Yang BZ, Chen S. Relationship between the catch of *Symlectoteuthis oualaniensis* and surface temperature and the vertical temperature structure in the South China Sea. Journal of fishery Science of China. 2016; 23:469–477. (in Chinese with English Abstract)

[7] Su L, Chen ZZ, Zhang P. Reproductive biology of purpleback flying squid (*Sthenoteuthis oualaniensis*) in the south-central South China Sea in spring and autumn. South China Fisheries Science. 2016; 12:96–102. (in Chinese with English Abstract)

[8] Zhang YM, Yan YR, Lu HS, Zheng ZW, Yi MR. Study on feeding and reproduction biology of purple flying squid sthenoteuthis in the western South China Sea. Journal of Guangdong Ocean University. 2013; 13:56–64. (in Chinese with English Abstract)

[9] Anuwat N, Aussanee M, Penkae D. Systematics and distribution of oceanic cephalopods in the South China Sea, Area III: Western Philippines. Proceedings of the SEAFDEC Seminar on Fishery Resources in the South China Sea, Area III: Western Philippines. 2000; 76–100.

[10] Worms J. World fisheries for cephalopods: a synoptic overview. FAO Fish Tech. 1983; 231:1–19.

[11] Siriraksophon S, Nakamura Y, Pradit S, Sukramongkol N. Ecological Aspects of Oceanic Squid, *Sthenoteuthis oualaniensis* (Lesson) in the South China Sea, In the Proceedings of the Third Technical Seminar on marine Fishery Resources Survey in the South China Sea, Area III: Western Philippines. Bangkok: Special Paper No. SEC/SP/41, SEAFDEC; 2000. pp. 101–117.

[12] Basir S. Proceedings of the SEAFDEC Seminar on Fishery Resources in the South China Sea, Area III: Western Philippines. In: Biological feature of an Oceanic Squid, *Sthenoteuthis oualaniensis*. Malaysia: SEAFDEC; 2000. pp. 135–147.

[13] Chen XJ, Zhao XH, Chen Y. Influence of El Niño/La Niña on the western winter–spring cohort of neon flying squid (*Ommastrephes bartramii*) in the northwestern Pacific Ocean. J Mar Sci. 2007; 64:1152–1160.

[14] Tafur R, Villegas P, Rabí M, Yamashiro C. Dynamics of maturation, seasonality of reproduction and spawning grounds of the jumbo squid *Dosidicus gigas*, (Cephalopoda: Ommastrephidae) in Peruvian waters. Fish Res. 2001; 54:33–50.

[15] Yang XM, Zhou YQ, Chen XJ, Tian SQ. Preliminary Study on Formation Mechanism of Fishing Ground of *Symlectoteuthis oualaniensis* in the Northwestern Indian Ocean Based on Marine Remote Sensing. Journal of Fisheries of China. 2006; 30:669–675. (in Chinese with English Abstract)

[16] Fan JT, Chen ZZ, Zhang J, Feng X. *Sthenoteuthis oualaniensis* fishing grounds analysis based on marine environment factors and different weight coefficient in the Zhongsha and Xisha Islands, South China Sea. South China Fisheries Science. 2016; 12:7–63. (in Chinese with English Abstract)

[17] YuW ChenX, YiQ ChenY, Zhang Y Variability of Suitable Habitat of Western Winter-Spring Cohort for NeonFlying Squid in the Northwest Pacific under Anomalous Environments. PLoS ONE. 2015; 10(4):e0122997 10.1371/journal.pone.0122997 25923519 PMC4414546

[18] Nigmatullin CM, Nesis KN, Arkhipkin AI. A review of the biology of the jumbo squid *Dosidicus gigas* (Cephalopoda: Ommastrephidae). Fish Res. 2001; 54:9–19.

[19] Hu QW, Chen XJ, Xu LQ, Yu J. Cluster analysis of tuna purse seine fishery in the Western and Central Pacific. Haiyang Xuebao. 2016; 38:66–75. (in Chinese with English Abstract)

[20] Yu J, Chen P, Tang D, Qin C. Ecological effects of artificial reefs in Daya Bay of China observed from satellite and in situ, measurements. Advances in Space Res. 2015; 55:2315–2324.

[21] Yu J, Tang DL, Imsang O, Yao LJ. Response of Harmful Algal Blooms to environmental changes in Daya Bay, China. Terr Atmos Ocea Sci. 2007; 18:1011–1027.

[22] Yu J, Tang DL, Yao LJ, Chen PM, Jia XP, Li CH. Long-term water temperature variations in Daya Bay, China using satellite and in situ observations. Terr Atmos Ocea Sci. 2010; 21:393–399.

[23] Tang DL, Liu YP, Hao XG, Wu CX, Wang CX, Yin YW. A newly-discovered historical map using both national boundary and administrative line to represent the U-boundary in the South China Sea (in Chinese). China Sci Bull. 2018; 63 10.1360/N972017-00440

[24] Kang L. Investigation and Study on Marine Fishery Resources in Xisha islands. Mar Fish. 2016; 12:64–66. (in Chinese with English Abstract)

[25] Wood S. Generalized Additive Models: an Introduction with R Boca Raton: Chapman Hall/CRC Press; 2006.

[26] Hinton MG, Maunder MN. Methods for standardizing CPUE and how to select among them. Col Vol Sci Pap ICCAT. 2004; 56:169–177.

[27] Gavaris S. Use of a multiplicative model to estimate catch rate and effort from commercial data. Ca J Fish Aquat Sci. 1980; 37: 2272–2275.

[28] Ver Hoef JM, Boveng PL. Quasi-poisson vs. Negative binomial regression: how should we model over dispersed count data. Ecology. 2007; 88:2766–2772. 18051645 10.1890/07-0043.1

[29] Burnham KP, Anderson R, Model D. Selection and multi-model inference: a practical information-theoretic approach. J Wildlife Manage. 2002; 67:175–196.

[30] Klemas V. Fisheries applications of remote sensing: An overview. Fish Res. 2013; 148:124–136.

[31] Chen XJ, Liu BL, Tian SQ, Qian WG, Zhao XH. Fishery biology of purple back squid, *Sthenoteuthis oualaniensis*, in the northwest Indian Ocean. Fish Res. 2007; 83:98–104.

[32] Tian SQ, Chen XJ, Yang XM. Distribution of fishing ground of *Symlectoteuthis oualaniensis* in the high seas of northern Arabia and its relationship with marine environmental factors. Bull Oceanol Limn. 2006; 51–57.

[33] Lin DM, Chen XJ. Distribution of fishing ground of *Symlectoteuthis oualaniensis* in the northwestern Indian Ocean and its relationship with sea surface temperature. Adv Mar Sci. 2006; 24:546–551.

[34] Campbell RA. CPUE standardization and the construction of indices of stock abundance in a spatially varying fishery using general linear models. Fish Res. 2004; 70:209–227.

[35] Ziegler PE, Frusher SD, Johnson CR. Space-time variation in catchability of southern rock lobster Jasus edwardsii, in Tasmania explained by environmental, physiological and density-dependent processes. Fish Res. 2003; 61:107–123.

[36] Bacha M, Jeyid MA, Vantrepotte V, Dessailly D, Amara R. Environmental effects on the spatio‐temporal patterns of abundance and distribution of sardina pilchardus and sardinella off the mauritanian coast (north-west africa). Fish Oceanogr. 2017; 8:12–25.

[37] Maunder MN, Punt AE. Standardizing catch and effort data: a review of recent approaches. Fish Res. 2004; 70:141–159.

[38] Bacha M, Jeyid MA, Vantrepotte V, Dessailly D, Amara R. Environmental effects on the spatio-temporal patterns of abundance and distribution of Sardina pilchardus and sardinella off the Mauritanian coast (North-West Africa). Fish Oceanogr. 2017; 26:282–298.

[39] Qian WG, Chen XJ, Liu BL, Tian SQ, Ye XC. The relationship between fishing ground distribution of Symlectoteuthis oualaniensis and zooplankton in autumn in the northwestern Indian Ocean. Mar Fish. 2006; 28:265–271.

[40] Wang JJ, Tang DL. Phytoplankton patchiness during spring intermonsoon in western coast of South China Sea. Deep Sea Res Part II. 2014; 101:120–128.

[41] Arkhipkin A, Mikheev A. Age and growth of the squid Sthenoteuthis pteropus, (Oegopsida: Ommastrephidae) from the Central-East Atlantic. J Exp Mar Biol Ecol. 1992; 163:261–276.

[42] Yan H. High-resolution climatic and environmental changes in the late Holocene of the Xisha Islands in the South China Sea University of Science and Technology of China 2012; 25–86. (in Chinese with English Abstract)

[43] Young RE, Hirota J. Description of Ommastrephes bartramii (Cephalopoda: Ommastrephidae) paralarvae with evidence for spawning in Hawaiian wate. Pacif Sci. 1990; 44:71–80.

[44] Bower JR, Seki MP, Young RE, Bigelow KA, Hirota J, Flament P. Cephalopod paralarvae assemblages in Hawaiian Islands waters. Mar Ecol Prog Ser. 1999; 185:203–212.

[45] Zhang ZM. El Niño and La Niña events and their effects on climate anomalies in the South China Sea and South China Sun Yat-Sen University 2001; 20–58. (in Chinese with English Abstract)

[46] Fu D, Tang D, Levy G. The impacts of 2008 snowstorm in China on the ecological environments in the Northern South China Sea. Geomatics Natural Hazards and Risk. 2017; 08:1–20. 10.1080/19475705.2017.1292559

[47] Zhang PQ, Jia XL, Wang YG. Anomalies of Ocean and General Atmospheric Circulation in 2008 and their Impacts on Climate Anomalies in China. Meteorology. 2009; 35:112–117. (in Chinese with English Abstract)

[48] Ye HJ, Kalhoro MA, Morozov E, Tang DL, Wang SF. Increased chlorophyll-a concentration in the South China Sea caused by occasional sea surface temperature fronts at peripheries of eddies. Int J Remote Sens. 2017; 2:1–16.

[49] Kuo NJ, Zheng Q, Ho CR. Response of Vietnam coastal upwelling to the 1997–1998 ENSO event observed by multisensor data. Remote Sens Environ. 2004; 89:106–115. 10.1016/j.rse.2003.10.009

[50] Yu J, Hu QW, Li CH, Zhang P, Mao JM. Relationship between the resource and environment factors in the Xisha-Zhongsha waters in spring. Haiyang Xuebao. 2017; 39:72–73, 10.3969/j.issn.0253–4193.2017.06.007(in Chinese with English Abstract)

